# Artificially intelligent nursing homes: a scoping review of palliative care interventions

**DOI:** 10.3389/fdgth.2025.1484304

**Published:** 2025-02-11

**Authors:** Isabel Ronan, Sabin Tabirca, David Murphy, Nicola Cornally, Mohamad M. Saab, Patrice Crowley

**Affiliations:** ^1^ School of Computer Science, University College Cork, Cork, Ireland; ^2^ Faculty of Mathematics and Informatics, Transilvania University of Brasov, Brasov, Romania; ^3^ School of Nursing and Midwifery, University College Cork, Cork, Ireland

**Keywords:** nursing, artificial intelligence, scoping review, palliative care, nursing home

## Abstract

**Introduction:**

The world’s population is aging at a rapid rate. Nursing homes are needed to care for an increasing number of older adults. Palliative care can improve the quality of life of nursing home residents. Artificial Intelligence can be used to improve palliative care services. The aim of this scoping review is to synthesize research surrounding AI-based palliative care interventions in nursing homes.

**Methods:**

A PRISMA-ScR scoping review was carried out using modified guidelines specifically designed for computer science research. A wide range of keywords are considered in searching six databases, including IEEE, ACM, and SpringerLink.

**Results:**

We screened 3255 articles for inclusion after duplicate removal. 3175 articles were excluded during title and abstract screening. A further 61 articles were excluded during the full-text screening stage. We included 19 articles in our analysis. Studies either focus on intelligent physical systems or decision support systems. There is a clear divide between the two types of technologies. There are key issues to address in future research surrounding palliative definitions, data accessibility, and stakeholder involvement.

**Discussion:**

This paper presents the first review to consolidate research on palliative care interventions in nursing homes. The findings of this review indicate that integrated intelligent physical systems and decision support systems have yet to be explored. A broad range of machine learning solutions remain unused within the context of nursing home palliative care. These findings are of relevance to both nurses and computer scientists, who may use this review to reflect on their own practices when developing such technology.

## 1 Background

As the world’s population continues to age rapidly, the number of older adults in need of care is increasing; the UN has indicated that by 2030 over 60 countries will have 2 million or more people aged 65 or older (1). Adequate nursing home care is needed to facilitate the personal and medical requirements of these people in a timely and efficient manner (2). In nursing homes where palliative care (PC) is implemented, improvements can be seen in the levels of clinical care received, reductions in hospitalizations, and enhanced family perceptions (3). There is a growing realization that new forms of care, such as PC, are needed to effectively care for our changing society (4).

While PC is becoming increasingly more popular, the field is complex, full of ethical issues and uncertainty surrounding the best time to provide such care (4). Education on the subject also needs improvement, with many burgeoning health professionals lacking adequate knowledge about such care (5, 6). Additionally, there are many interpretations of what comprises the discipline across cultures, countries, and clinical norms (7). This situation leads to confusion about what exactly constitutes PC (8). In this review, we describe PC as an approach to care which emphasizes relief from suffering and improved well-being, especially in the terminal stage of illness or the final period of life (6–8).

Technology can be utilized to provide health professionals with care tools, while also creating a higher quality of life for older adults. Artificial Intelligence (AI) has already proven itself to be useful within the context of PC (9). AI is a broad field encompassing all systems which exhibit human-like intelligence (10). Often used synonymously with AI, machine learning (ML) is a subset of the overarching discipline (10). ML involves “learning” on data while using computational algorithms (10). While applications of AI have been explored and consolidated within the context of general nursing care (11), AI interventions in nursing homes have yet to be reviewed in depth.

As PC is a highly sensitive area, including those who will use proposed AI systems in their development would have quite an influence over system designs. Feedback from healthcare professionals on the utility of using such technology and the ethics of AI analysis may ensure that AI interventions are in-keeping with palliative principles. PC is a subtle area, full of sensitivities; the involvement of system end-users in the creation of these tools is paramount to their success.

AI and ML technologies could prove to be quite useful in nursing home settings. There has already been work on the impact of such tools in geriatric clinical care (12), revealing that AI can be used for early detection and prevention of severe illnesses. Algorithm choice can increase the accuracy of results, reduce computational load, and make such tools more lightweight and easy-to-use. Deep Learning (DL), a subsection of machine learning, has also shown extensive capabilities in the field of geriatric clinical care (12).

We chose to undertake a scoping review due to the breadth and depth of our topic. In this paper, we will consolidate past work, examine emerging areas of exploration, and identify knowledge gaps. Due to the wide range of possible interventions and studies on the subject, a scoping review was deemed appropriate. The objectives of this review are to explore what AI technologies are used in palliative nursing home research, to discern what types of ML algorithms and AI methods are used in palliative nursing home studies, and to assess the role of end-users in palliative technological developments and assessments.

To the best of the authors’ knowledge, our review is the first of its kind to focus exclusively on this topic. As a result, a broad range of PC interventions and technologies are considered. There does not seem to be much analysis of data accumulated in nursing homes as of yet, whether in the form of data science techniques or AI interventions (13). In this paper, we will investigate how, even if limited, nursing homes have adopted AI specifically for PC.

This review’s research questions were chosen to encompass the breadth of material which could be considered AI-related within palliative nursing home contexts.

1. What palliative systems or strategies incorporating AI have been designed for, used in, or studied in nursing home settings?
2. What AI techniques are used within palliative technologies in nursing home settings?
3. How do relevant projects include health professionals and/or nursing home residents and/or their families in project design, deployment, or testing processes?

Question 1 aims to capture the broad range of palliative approaches that can be considered when using AI. Question 2 concentrates on the different AI techniques used to approach PC. Algorithms, data inputs, and data outputs will also be examined. Question 3 addresses key stakeholder involvement. User-centered testing procedures associated with AI-based interventions will also be explored.

This review’s main contribution to the literature is an up-to-date collation of artificially intelligent nursing home PC studies. This review could be of use to AI researchers and developers, along with decision-makers in nursing homes. The rest of this paper is divided into the following sections: methods, results, discussion, and conclusions.

## 2 Methods

This review was carried out during the months of April and May 2024. The Parsifal review and Zotero reference manager softwares were used for content organization (14, 15). Specifically, Parsifal was used to record screening decisions and extracted data points. Zotero was used to store and read all relevant materials. All searching and screening was predominantly carried out by the first author, with guidance from other authors. Three of these authors also contributed to the study selection process.

### 2.1 Operational definitions

Our research questions are framed around the use of “palliative systems or strategies” in nursing homes. These terms need clarification in order to be used effectively in this review. When we refer to a “palliative system” we are referring to a technological system designed specifically for PC or built with a palliative module included. We do not refer to general systems which do not involve technology. When we refer to “palliative strategy,” we are also referring to some form of technological process. As this is a broad review, we were not to be too prescriptive when choosing these terms. However, we insisted that there must be a technological element involved in any system or strategy we selected for inclusion in this review.

### 2.2 Protocol and registration

We drafted our protocol using the Preferred Reporting Items for Systematic Reviews and Meta-Analyses Extension for Scoping Reviews (PRISMA-ScR). We also incorporated elements of a pre-existing systematic review guide for computer science research by Carrera-Rivera et al. into our work (16). This guide outlines an algorithmic approach to the review process, which we found useful for structuring our own research. We did not register our protocol.

### 2.3 PICOC criteria

The Population, Intervention, Comparison, Outcome, and Context (PICOC) criteria were used to define the specificity and focus areas of the review. These criteria were carefully chosen to encompass nursing homes, PC, and AI research. Table 1 outlines the different criteria chosen along with their associated keywords and synonyms.

**Table 1:** PICOC criteria.

Criterion	Keyword	Synonyms
Population	Nursing home	Residential care home, care home, assisted living facility, convalescent home, convalescent hospital, old folks home, old peoples home, rest home, retirement facility, retirement home, aged care facility, skilled nursing facility
Intervention	Artificial intelligence	AI, expert system, intelligent retrieval, knowledge engineering, machine learning, natural language processing, neural network
Comparison	Paper-based methods	Manual data, clinical judgement
Outcome	Palliative care	Palliation, palliative therapy, palliative treatment, end-of-life care, hospice care, EoLC, palliative medicine, comfort care, supportive care
Context	Healthcare tools	ICT technology, HICT, healthcare technology, health technology, health systems, health tools

These criteria were subsequently used to focus our digital library searches.

### 2.4 Eligibility criteria

Eligibility criteria were defined in order to screen articles from initial searches. Articles were screened based on their titles and abstracts. Table 2 outlines the chosen criteria.

**Table 2:** Eligibility criteria.

Criterion	Inclusion	Exclusion
Period	From January 2018 to April 2024	Prior to January 2018
Language	Articles in English	Articles not in English
Material type	Peer-reviewed journal and conference articles	Reports, policy literature, working papers, newsletters, government documents, speeches, IEEE Early Access articles, articles from books, non-peer reviewed materials, conference abstracts, literature reviews
Quality	Q1 journal articles, Q2 journal articles, high-rank conferences (e.g., A*, A, B)	Q3 journal articles, Q4 journal articles, low-rank conferences (e.g., C), unranked conferences
Accessibility	Open access, university-provided access, alternative access options	Inaccessible, purchase required
Relevance	Articles relevant to one or more research questions	Articles not relevant to any research questions

Material within the last seven years (i.e., January 2018 to April 2024) was chosen based on the constantly fluctuating technological landscape. This time period broadens the scope of the review, while not including papers that are potentially out-of-date. Articles were limited to those in English due to resource constraints; obtaining adequate translations for other languages was unfeasible. Only peer-reviewed academic articles from journals and conferences were included to ensure high-quality materials are discussed in our findings; this criterion strengthens the validity of this review’s results. Other types of material were not considered due to difficulties in proving the quality of the same. Additionally, other literature reviews were excluded as they are secondary sources of information.

Journal articles within the first or second impact factor quartiles were considered within this review to ensure that high quality studies are discussed in our findings. All quartile-related information on journals was found in the Journal Citation Reports website (17). All reputable conference articles were included; the ICORE Conference Portal was used for computer science conferences (18). Relevant healthcare conferences were assessed by one of the nursing-related authors involved in the review to determine quality and subsequent inclusion or exclusion. Inaccessible or costly articles were not included in this review. Articles had to be relevant to at least one of the research questions to be considered for inclusion.

### 2.5 Information sources

Digital libraries were selected for searching based on their relevance to the problem area. Some of these repositories were also chosen based on their appearance in other systematic literature reviews on similar subjects (11). Namely, the ACM Digital Library, IEEE Digital Library, ISI Web of Science, Science Direct, Scopus, and Springer Link were used.

Additionally, all authors contributed to the search for materials by hand-picking a selection of studies they deemed potentially relevant to the review; these studies were included in subsequent filtering processes to ensure their relevance and quality. As these hand-picked studies were chosen by authors with research backgrounds in either computer science or nursing, we incorporate both health and computing perspectives into the search process.

### 2.6 Search

**Figure 1:**
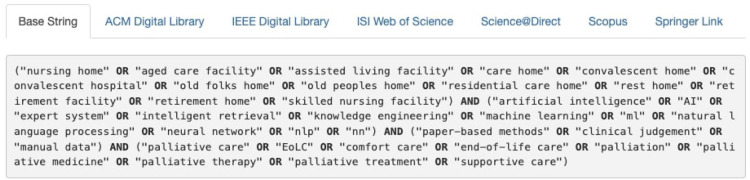
Base search string.

Each library has its own search engine features, facilitating advanced search strings and keyword usage. The keywords used were derived from the PICOC criteria. Search queries all followed a similar structure involving primary keywords such as “nursing home,” “palliative care,” and “artificial intelligence.” Search strings were adjusted from a base string to facilitate an accurate search of each database. Figure 1 shows the base search string as displayed in Parsifal (14).

Results of searches were filtered by year, content type, and accessibility. Subsequently, the results were exported as BibTeX files and imported into the Parsifal software for screening.

### 2.7 Selection of sources of evidence

Each article was read and assessed using a four-question quality instrument; these questions were inspired from example questions provided by Carrera-Rivera et al. (16). Each question was answered using a 3-point answer scale; all answers were then summed to create an overall score for each article. A cut-off threshold was then outlined to ensure only high-quality articles were included in the findings of this review. Articles that scored higher than 2 were included and articles that scored lower than or equal to 2 were excluded. The aforementioned quality instrument is outlined in Table 3.

**Table 3:** Quality instrument.

Questions	Answers
Is the study relevant to my research?	Yes (+1)
Is the research methodology clearly outlined?	Partially (0)
Are the study results clearly described in the paper?	No (−1)
Is there a clear discussion of the limitations of the study?	

The concept of relevance, as used in our quality instrument, involved a couple of key considerations. These considerations are outlined below:

• Material must have a nursing-home focus or at least a dedicated discussion of nursing home settings.
• Material must explicitly mention PC or have a section discussing the same.
• Material must explicitly mention AI or at least have a section discussing AI or AI-based applications.
• Material does not focus on one disease specifically (e.g., residents with dementia only), but can target a specific illness if the entire nursing home population is included in the intervention (e.g., MCI or frailty).

### 2.8 Data charting process and data items

**Figure 2:**
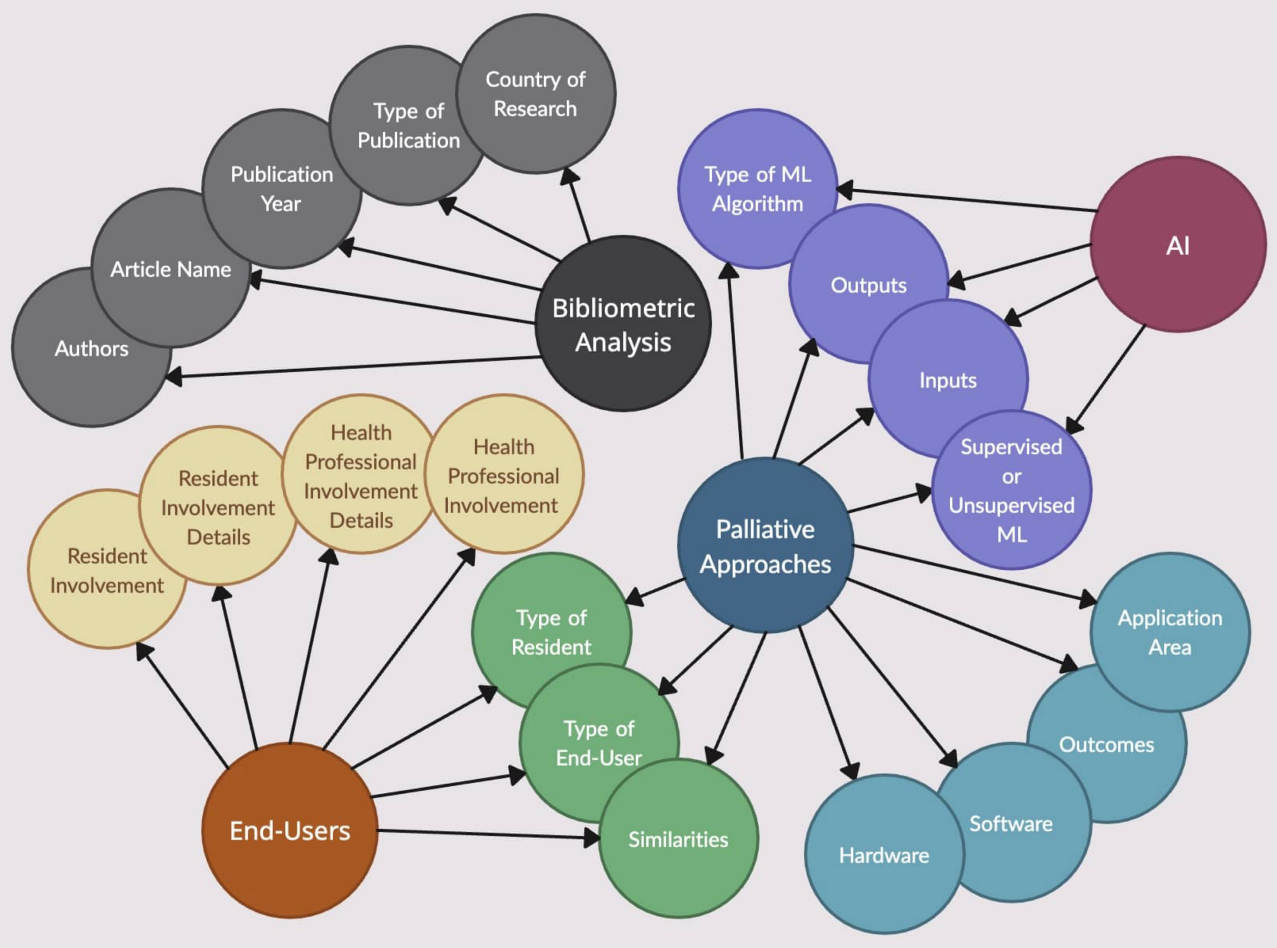
Data extraction form visualisation.

A data extraction form was created using Parsifal in order to collect the key features of each article (14). These key features were used during subsequent analysis. Features collected included nurse involvement, data inputs and outputs, and palliative outcomes. A test sample of 5 papers was used to assess the quality of the form; standardized terms were then chosen and used in subsequent data recording. For example, if a study mentioned the use of an Android smartphone or an iPhone, we simply recorded “phone” in the form. All form elements are outlined in Figure 2.

### 2.9 Synthesis of results

Data was processed using Python and Jupyter Notebooks to allow for easy visualization of results. Additionally, by using a code-based synthesis process, we ensure our analysis can be repeated. Various data points from the data charting process were collated and visualized in graphs.

## 3 Results

**Figure 3:**
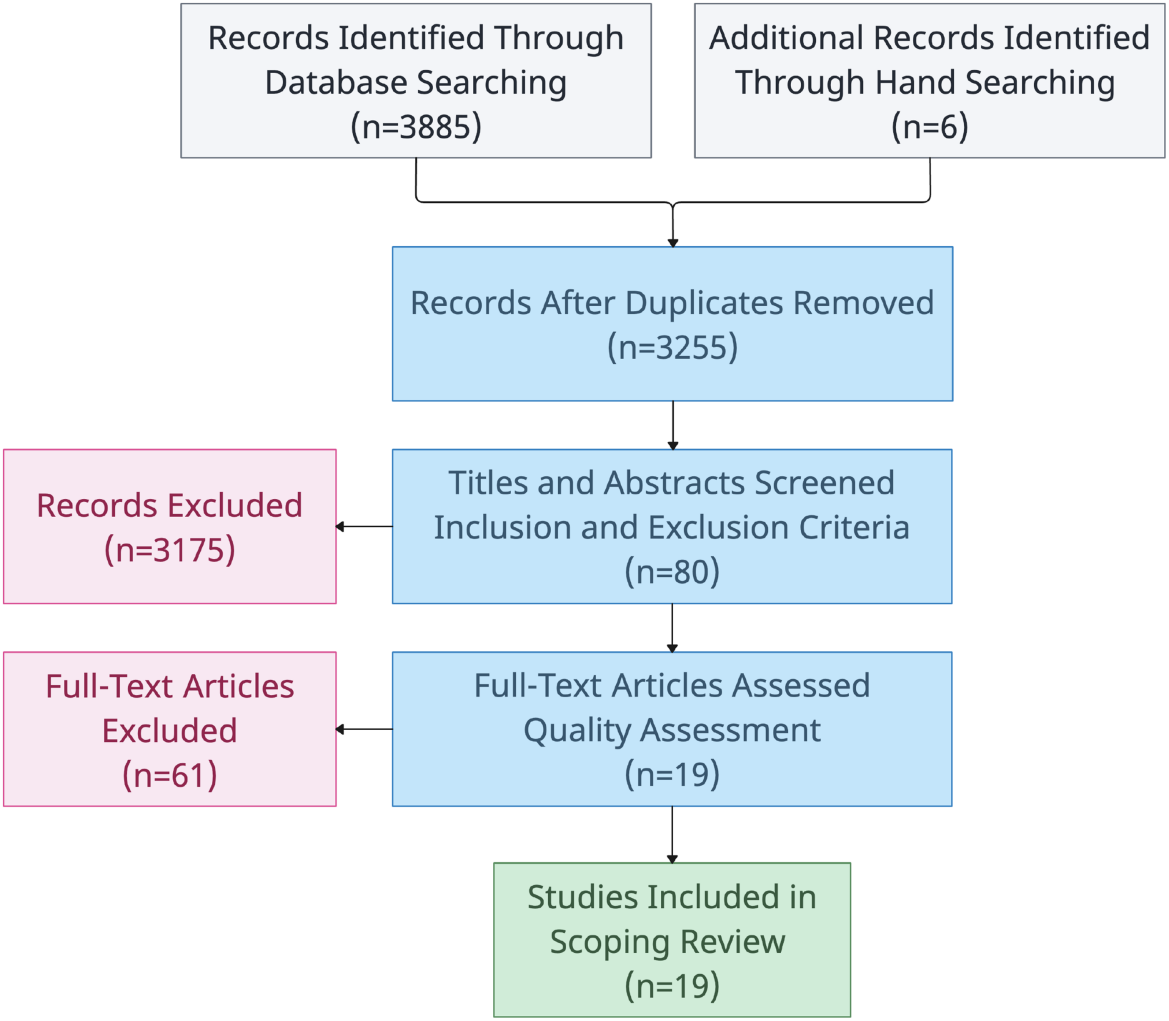
Article selection process.

The search process along with its associated results are outlined in a PRISMA-style flowchart in Figure 3 (19). Additionally, a summary of material sources is outlined in Table 4. A total of 636 duplicates were removed after the initial search process.

**Table 4:** Material sources.

Source	Number of results
ACM digital library	33
IEEE digital library	1,953
Science direct	111
Scopus	1,159
ISI web of science	3
Springer link	626
Hand-picked	6

The number of excluded materials from the title and abstract screening stage are outlined in Table 5. Most journal articles were excluded due to their quartile ranking or relevance at this stage in the review process. By the end of the title and abstract screening process, 80 articles were deemed suitable for full-text screening and quality assessment. 61 of these articles were excluded during quality assessment, leaving 19 articles eligible for data extraction and subsequent inclusion in this review.

**Table 5:** Title and abstract screening results.

Reason	Number excluded
Period	2
Language	40
Material type	44
Quality	1,048
Accessibility	0
Relevance	2,041

### 3.1 Bibliometric analysis

#### 3.1.1 Authors

All papers were authored by different academics with no repeated names appearing between papers. Therefore, no researchers stand out as being particularly prolific within the field of palliative nursing home AI interventions. This circumstance may reflect the fragmented nature of the field.

#### 3.1.2 Research topics

**Figure 4:**
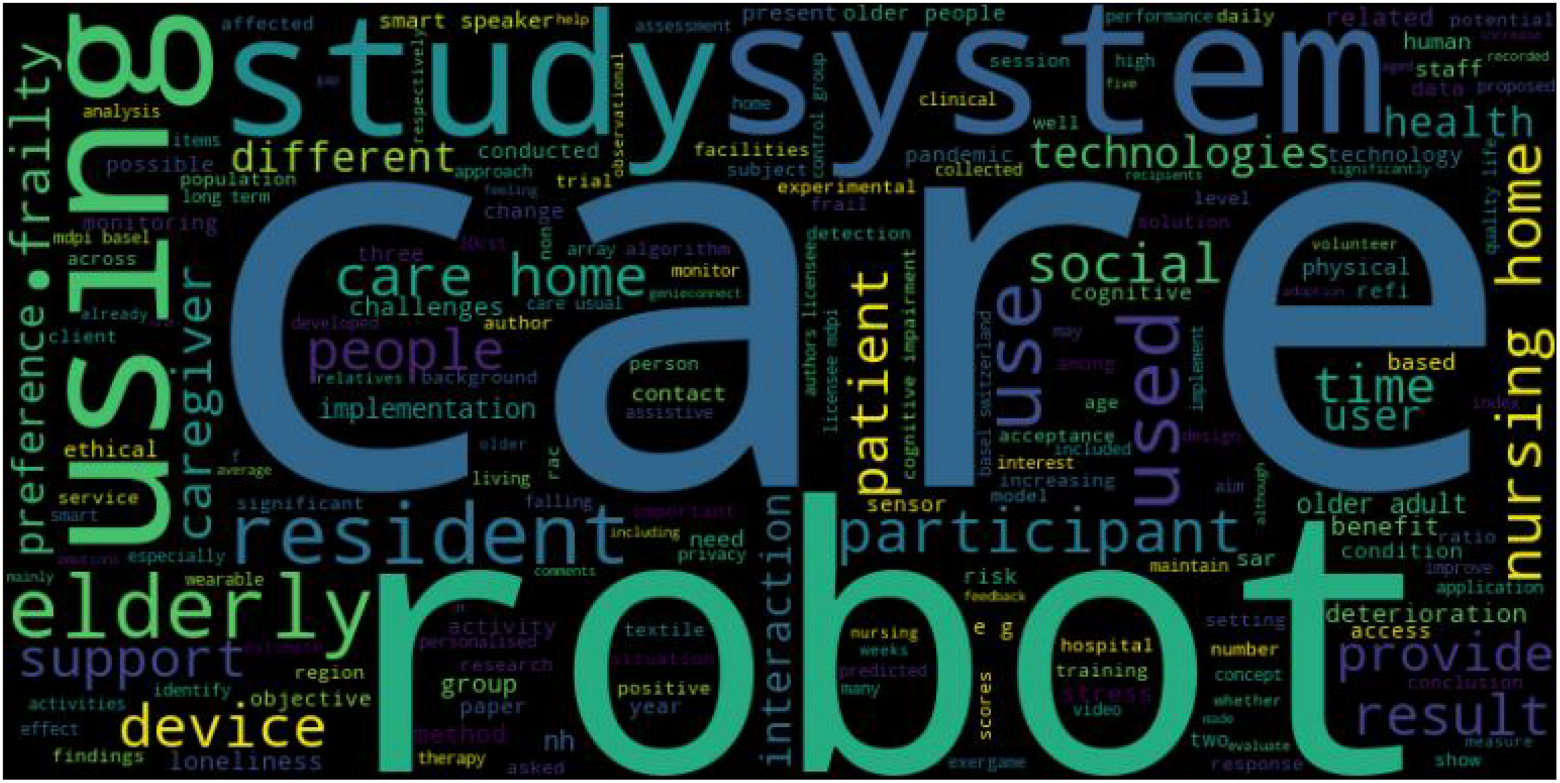
Abstract keyword word cloud.

As shown in Figure 4, abstract analysis reveals that the ten most common words found across studies were “care,” “robot,” “system,” “using,” “study,” “elderly,” “resident,” “use,” “used,” and “participant.” Words such as “care,” “robot” and “elderly” reveal potential focus on robot usage in older adult care settings. Additionally, words such as “study,” “system,” “participant,” “using,” “use,” and “used” suggest that the literature may focus on practical applications of systems and user responses to the same.

**Figure 5:**
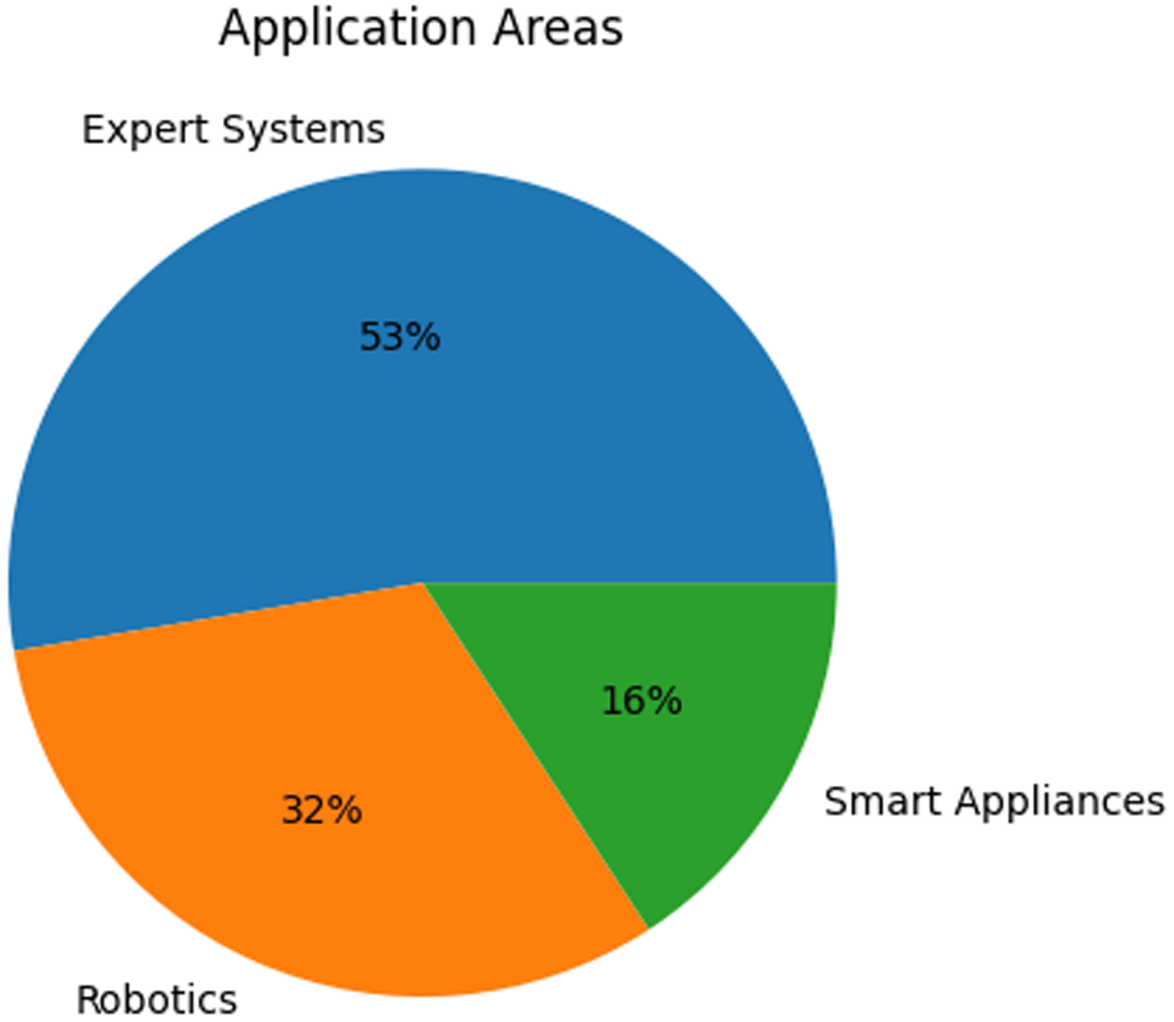
Application areas.

Analysis of application areas obtained during the data extraction process confirm the suppositions outlined above. Figure 5 shows that the majority of the studies focus on the use of expert systems or decision support (DS) systems in nursing homes, while other research involves the smart devices and robotics which comprise intelligent physical (IP) systems.

#### 3.1.3 Publication year

**Figure 6:**
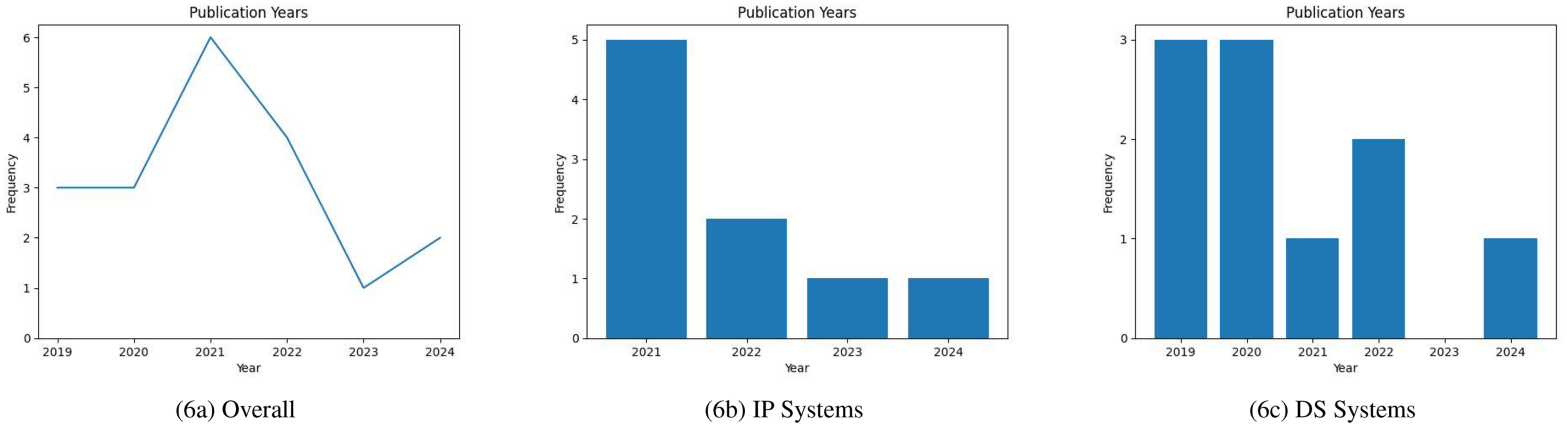
(**a**) Overall publication years; (**b**) IP systems publication years; (**c**) DS systems publication years (incomplete data for 2024).

Figure 6a conveys the temporal publishing landscape surrounding our research questions. Overall trends show that there was a significant spike in publications in 2021. As shown in Figure 6b there was a sudden surge in interest in IP technology in 2021. This spike in interest could be attributed to the COVID-19 pandemic, wherein non-contact forms of care were prioritized to preserve patient safety. In recent years, there seems to be interest in both DS systems and IP systems.

#### 3.1.4 Location

**Figure 7:**
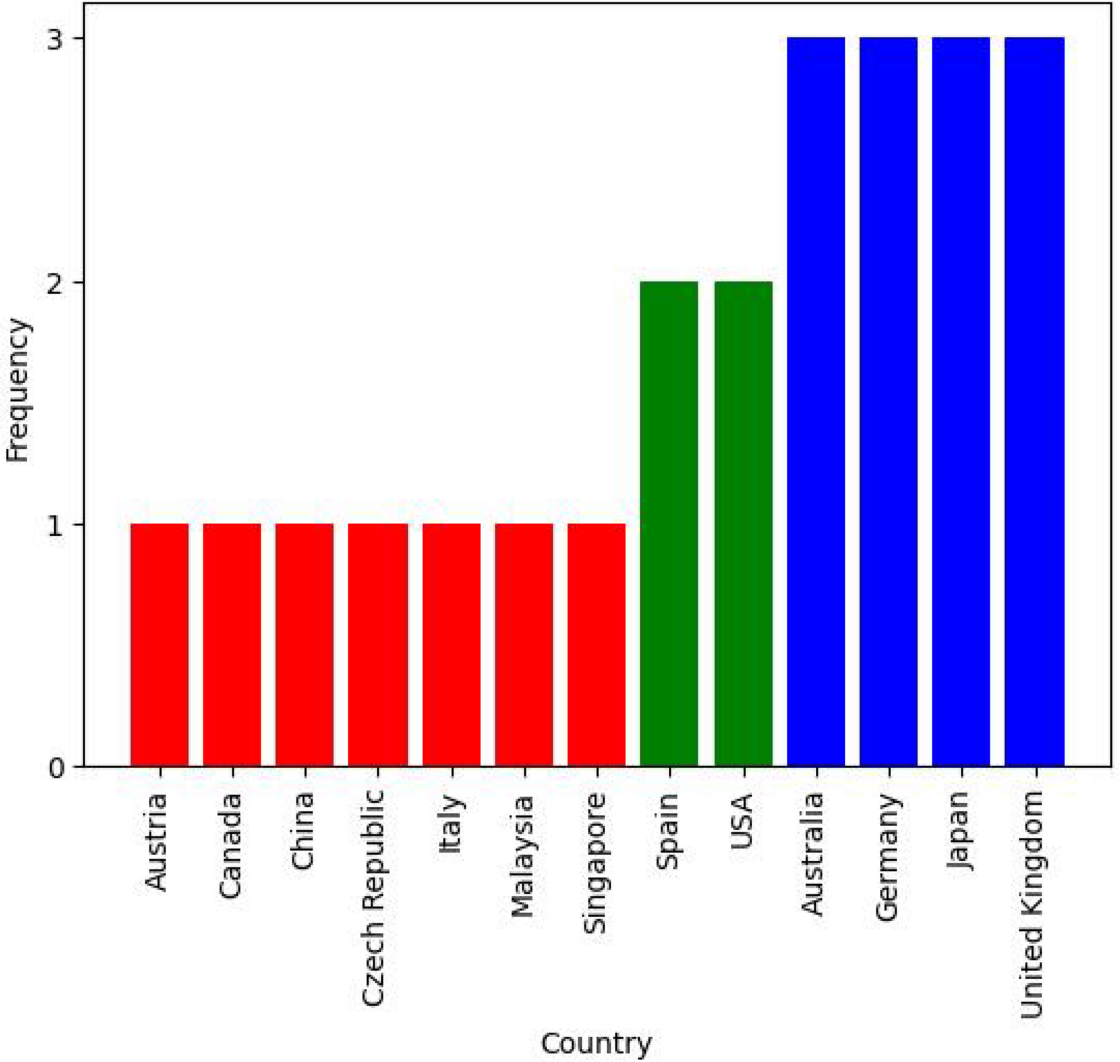
Research countries.

As outlined in Figure 7, the majority of studies in this review are from Europe. Asia, Oceania, and North America also feature in the literature. The proportion of research outputs per continent is fairly equal for both IP and DS systems, indicating that both subcategories are represented well geographically. There is also a pronounced absence of studies from Africa and South America; from this absence, one can posit that either the PC needs of people from these locations are not adequately addressed or there is not comparable research activity in these regions. This absence reflects findings from other palliative reviews focusing on these continents (20, 21).

### 3.2 Results of individual sources of evidence

Table 6 outlines all relevant outcomes data for each source of evidence.

**Table 6:** Data extraction table.

Paper	Year	Country	Hardware	Software	Outcomes	Resident	Algorithm	Input	Output	Resident role	Caregiver role
Delmastro et al. (22)	2020	Italy	ECG sensor; EDA sensor; phone; cycle ergometer	Weka; mobile app	Stress detection	Frail	Random forest; AdaBoost	Sensor data	Stress classifier	Database	N/A
Rettinger et al. (23)	2024	Austria	Socially assistive robots	N/A	Improved cognitive skills; alleviating loneliness	Any	N/A	Voice; touch	Audio-visual stimuli	User experience evaluation	Participant selection; test environment arrangement
Hayashi et al. (24)	2024	Czech Republic; Malaysia; Spain; Japan	BCG sensor	N/A	Decline detection	Any	Linear regression; one-class classifier; K-means clustering	Sensor data	Decline classifier	Database	N/A
Sarwar et al. (25)	2022	Australia	N/A	N/A	Decline detection	Frail	Logistic regression	Clinical data	Frailty status	Database	Development; validation
Oatley et al. (26)	2021	Australia	Smart textile; touch sensor	N/A	Cognitive assessment	Cognitively Impaired	N/A	Touch	Audio-visual stimuli; cognitive assessment	N/A	N/A
Wilson et al. (27)	2022	UK	Socially assistive robots	N/A	Alleviating loneliness	Any	N/A	Voice; touch	Audio-visual stimuli	User experience evaluation	Participant selection
Edwards et al. (28)	2021	UK	Smart speaker	Alexa	Alleviating loneliness; increasing independence	Any	N/A	Voice	Audio-visual stimuli	Database	Participant selection; test environment arrangement; user experience evaluation
Davitt and Brown (29)	2022	USA	Smart speaker	Alexa	Alleviating loneliness; well-being improvement	Any	N/A	Voice; touch	Audio-visual stimuli	Database	Participant selection; user experience evaluation
Tulsulkar et al. (30)	2021	Singapore	Socially assistive robots	N/A	Improved cognitive skills; alleviating loneliness; well-being improvement	Any	Convolutional neural network; linear regression	Voice; image	Audio-visual stimuli	Database	Database
Miranda-Duro et al. (31)	2021	Spain	Step sensor; sleep sensor	SPSS	Decline detection	Any	Logistic regression	Sensor data; clinical data	Fall risk	Database	N/A
Follmann et al. (32)	2021	Germany	Socially assistive robots	SPSS	Alleviating loneliness	Any	N/A	Voice; touch	Audio-visual stimuli	User experience evaluation	Participant selection
Tateno et al. (33)	2020	Japan	Infrared array sensor	N/A	Decline detection	Any	Recurrent neural network; convolutional neural network	Image	Fall risk	N/A	N/A
Becker et al. (34)	2020	Germany	Weighing sensor	N/A	Decline detection	Any	Decision tree	Sensor data	Fall risk	Database	N/A
Ambagtsheer et al. (35)	2019	Australia	N/A	SPSS; excel	Decline detection	Frail	Logistic regression	Clinical data	Frailty status	Database	Database
Papadopoulos et al. (36)	2021	UK; Japan	Socially assistive robots	CARESSES	Well-being improvement; alleviating loneliness	Any	N/A	Voice; image; touch	Audio-visual stimuli	User experience evaluation	Participant selection
Schönmann et al. (37)	2023	Germany	Socially assistive robots	N/A	Resident perspective tool	Any	N/A	N/A	N/A	N/A	N/A
Bourbonnais et al. (38)	2019	Canada	Camera; computer; phone	Mobile app; excel	Well-being improvement	Any	N/A	Image	Fall risk	N/A	User experience evaluation
Gannod et al. (39)	2019	USA	N/A	N/A	Well-being improvement	Any	Logistic regression	Clinical data	Custom recommender	Database	Participant selection
Tang et al. (40)	2022	China	Sensor; computer; phone	N/A	Decline detection	Any	Dual fuzzy logic; Case-based reasoning	Sensor data; clinical data	Decline classifier	Database	System development; test environment arrangement; user experience evaluation

#### 3.2.1 Nursing home data availability

Regarding IP systems, most of the studies were based on resident observations; thus data collected in these studies is reported within the material itself, and, in the majority of cases, is the basis of the publication. There was only one IP study which focused on the development of a system as opposed to resident observations (26). This study did not include actual nursing home residents, their caregivers, or their families in any part of their research process and thus data collected for this study does not come from nursing homes.

Within the context of data-heavy DS systems, one study had system-specific data freely available to download and use (24); this sensor data is not generally applicable to a wide variety of nursing home settings. One study did not explicitly mention any system-reliant data collected as part of their research. Instead, they focused on caregiver perspectives to hypothetical technologies; similarly to the majority of IP literature, this study’s data is reported within the material itself (38). Four DS system studies did not disclose the datasets used in their studies, their data could not be found, or their data did not come from nursing homes (31, 33, 39, 40). Four DS studies were asked about potential data access (22, 25, 34, 35). Two of these studies explicitly mention the availability of their data upon reasonable request (25, 34). Three kind responses were obtained, but data was not available in the two aforementioned cases (25, 34). One response indicated that data was available, provided research to be undertaken using the same was appropriate (22). Overall, data from these nursing home studies is not openly available and quite difficult to obtain.

### 3.3 Synthesis of results

#### 3.3.1 Types of systems or strategies

The literature exhibits a strong focus on IP systems along with DS systems. IP systems are entities capable of interacting with the physical world using sensors, actuators, processors or a combination of the same; these systems can be cognizant of their environments, perform tasks, and make decisions based on user input (41). DS systems utilize ML to analyse data and provide informed recommendations for end-users; these systems use domain-specific information to create data-driven insights for decision-makers (42).

**Figure 8:**
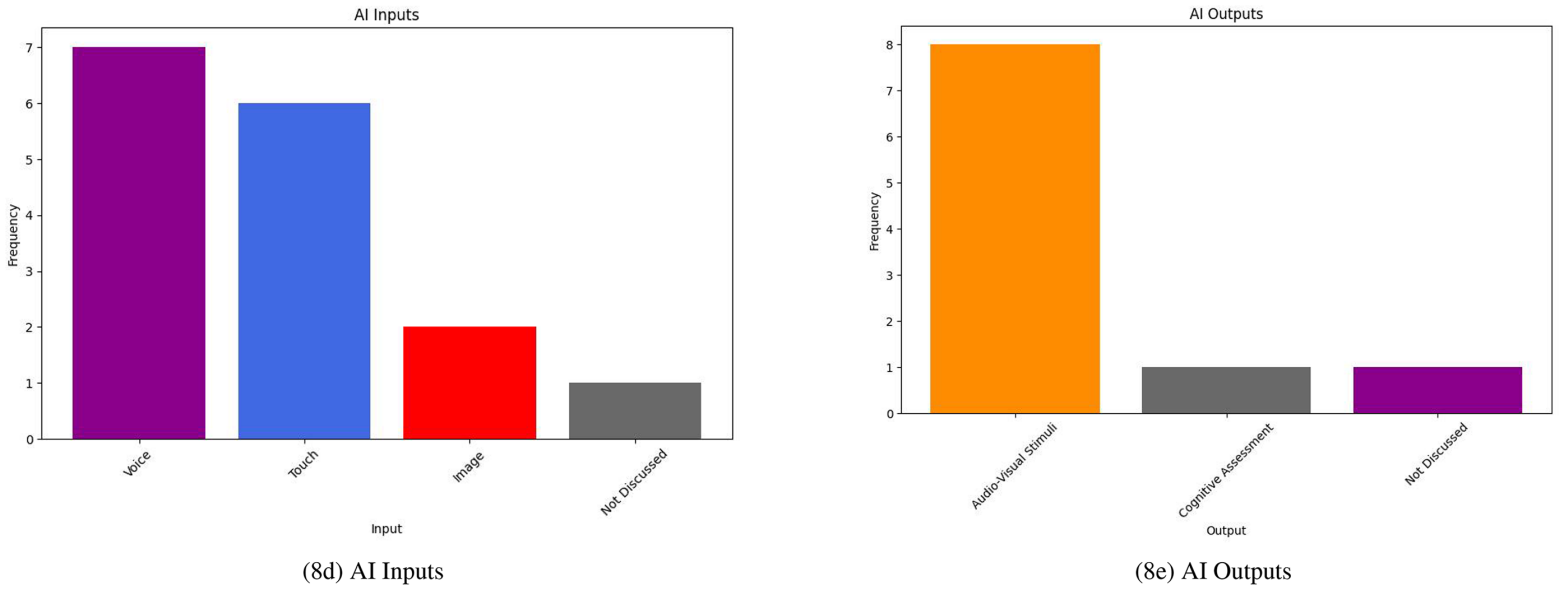
IP systems I/O.

Many of the IP systems in this review use socially assistive robots (SARs) in their research (23, 27, 30, 32, 36, 37). Smart speakers are used in a few studies to provide means of communication for residents, caregivers, and family members (28, 29). One study describes a smart textile for the nursing home use case (26). As shown in Figure 8 the majority of these systems focused on providing audio-visual stimuli for their users and accepted both voice and touch as the primary forms of input.

**Figure 9:**
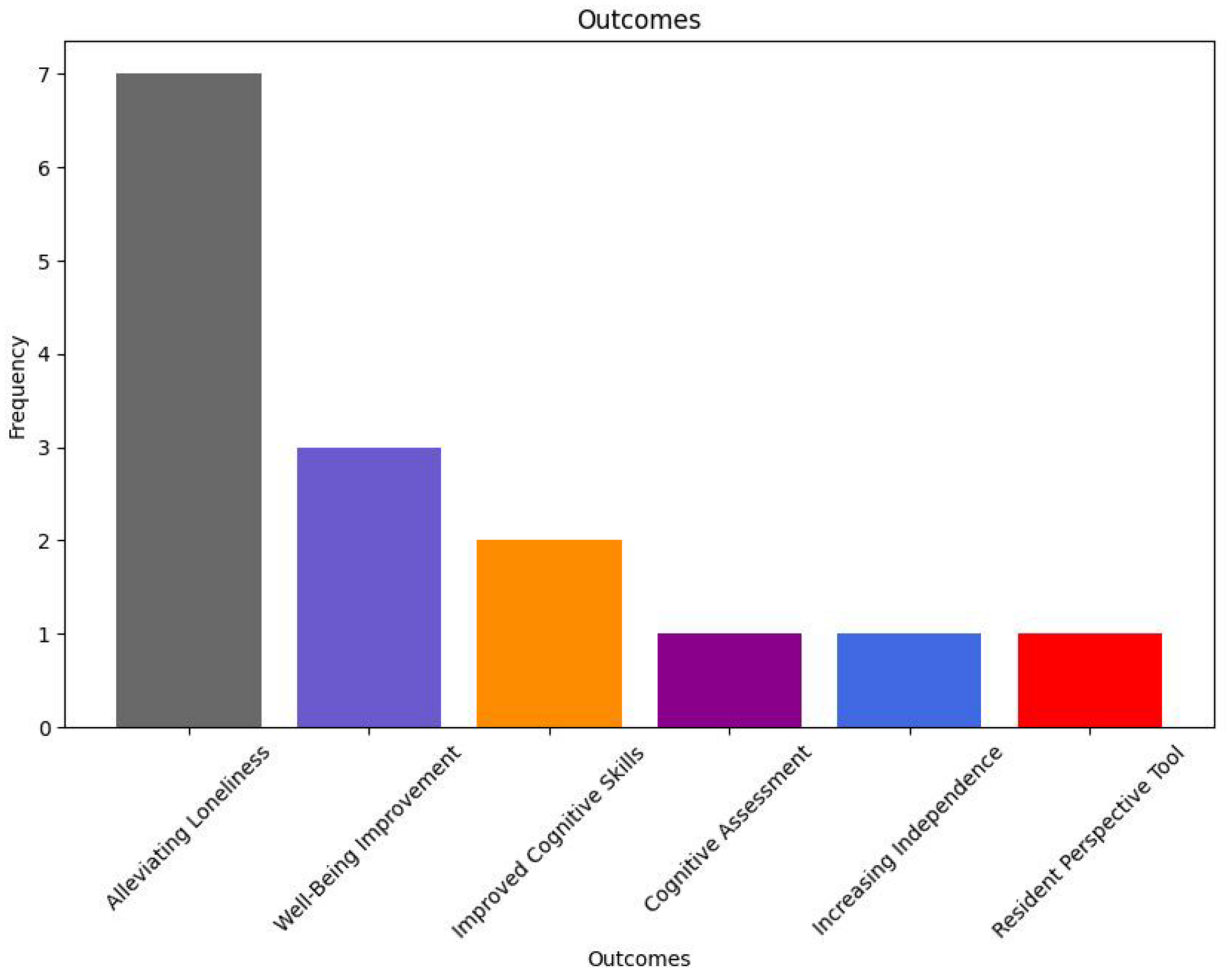
IP system palliative outcomes.

Figure 9 conveys the fractured landscape surrounding palliative outcomes of IP studies. As in the examples outlined above, many relevant studies discussed the potential social benefits of such systems. There seems to be a focus on research surrounding loneliness alleviation (23, 27–30, 32, 36). Well-being improvement and improved cognitive skills are also discussed in a few studies (23, 29, 30, 36). However, no clear palliative definitions are discussed in any of the relevant studies, making fundamental comparison between them difficult.

**Figure 10:**
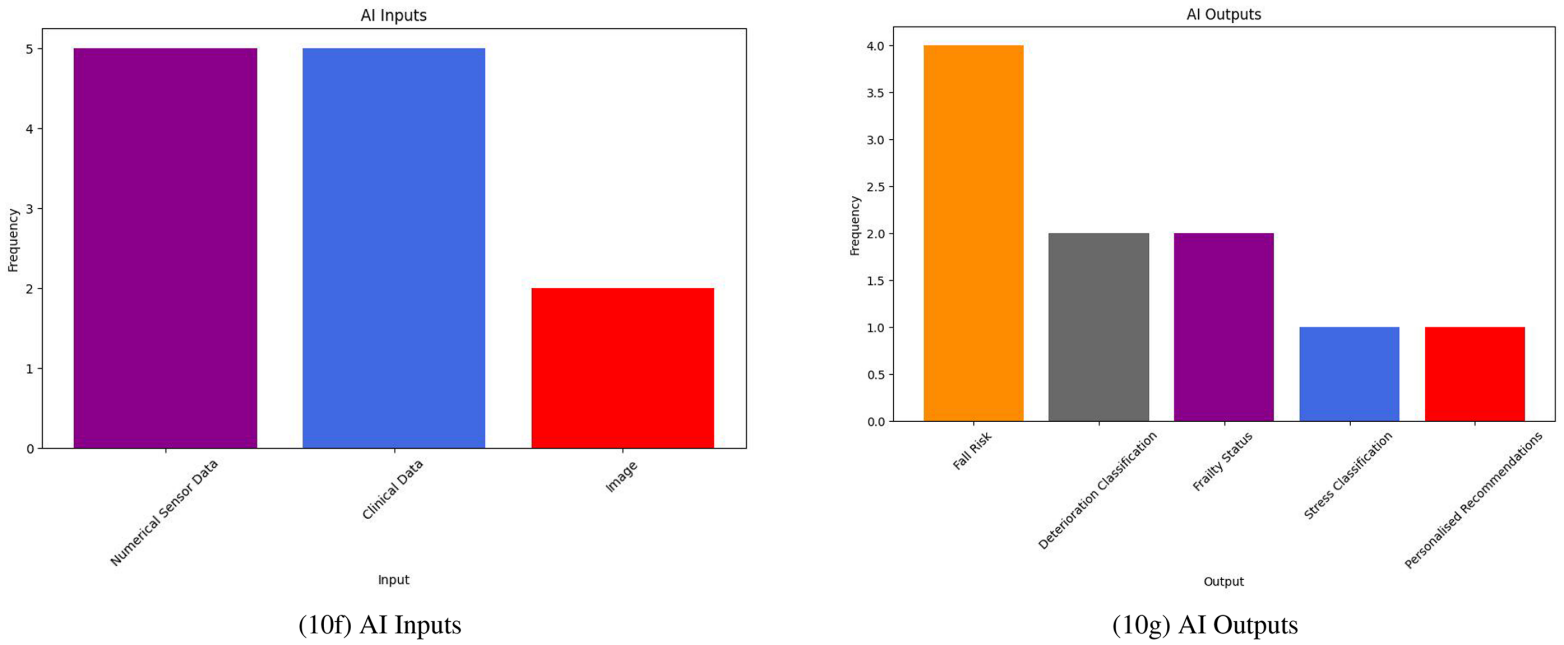
DS systems I/O.

Many of the DS system studies included the use of some form of sensor-based dataset to create algorithmically-driven solutions; these sensors generated either some form of numerical data or image which could be used to train ML algorithms. As outlined in Figure 10, all studies discussed some form of ML inputs and outputs in their work. Inputs to the majority of these systems were either sensor data or clinical data; one study included a mixture of the two (31).

A wide variety of different sensors were used in the DS systems from the literature, such as weight, ballistocardiogram (BCG), electrocardiogram (ECG), electrodermal activity (EDA), step and sleep sensors (22, 24, 31, 34). Images extracted from infrared array sensors or cameras are also mentioned in a few DS studies (33, 38).

All DS studies used different forms of sensor and clinical data to infer patient outcomes. Clinical variables widely varied from country-specific assessments to collected data from patient-level tools (25, 31, 35, 39). Sarwar et al. includes the use of progress notes, a common, but somewhat underutilized, form of data collection in nursing homes (25, 43). Ambagtsheer et al. developed their findings using the Australian Aged Care Funding Instrument (ACFI) (35). However, this tool is not applicable to nursing homes outside of Australia.

**Figure 11:**
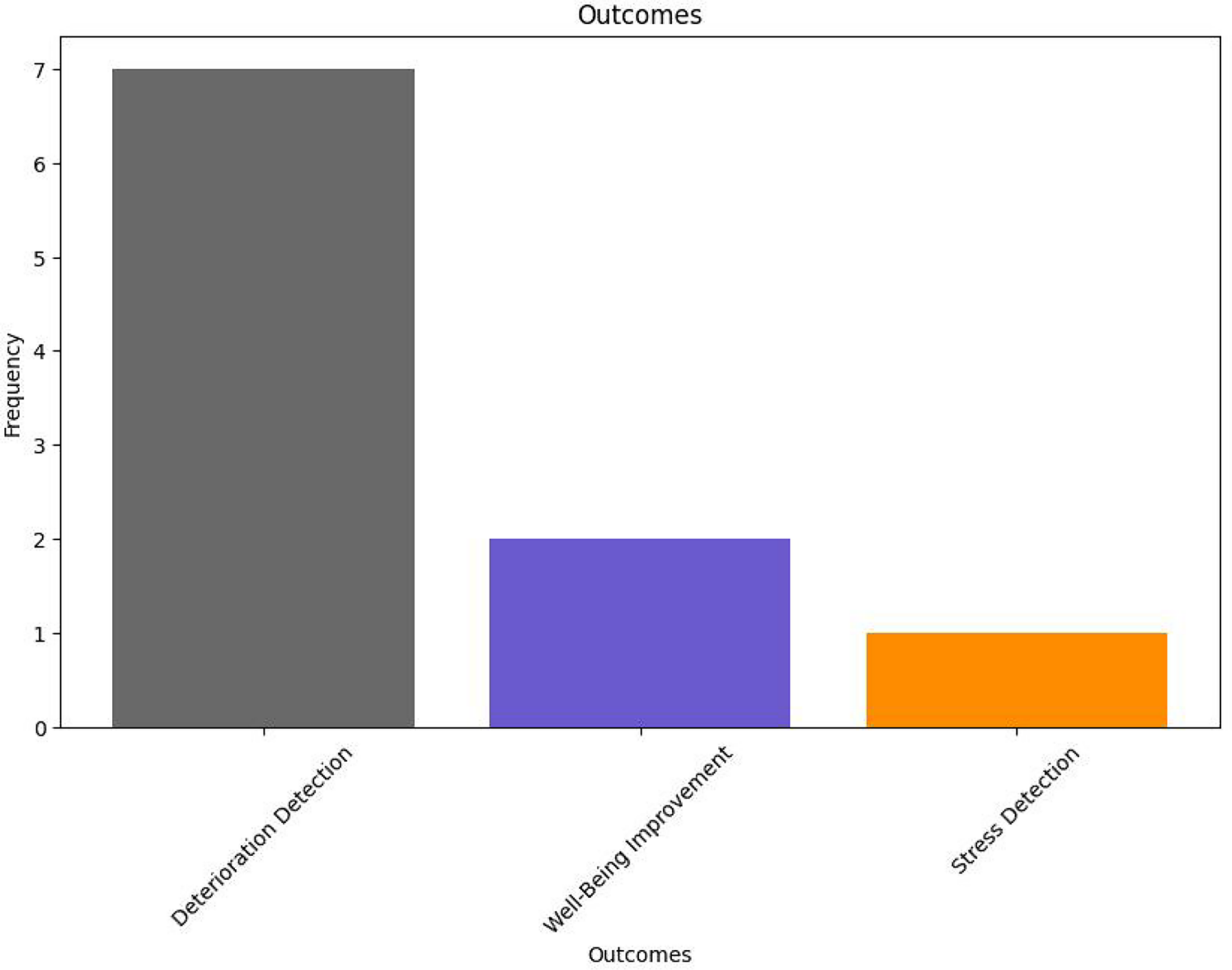
DS system palliative outcomes.

As shown in Figure 11, DS systems feature a much narrower range of palliative outcomes than IP systems. Most DS system interventions placed emphasis on deterioration detection as their primary palliative outcome (24, 25, 31, 33–35). Similarly to IP systems, a few DS system studies also considered general well-being improvement as their primary goal (38, 39). Furthermore, in line with findings from IP systems, no studies define exactly what PC means within the context of their research. All studies focus on a more broad view of the same.

#### 3.3.2 AI techniques

Most IP system papers did not discuss the AI techniques used in their research. Only one reported the use of a convolutional neural network (CNN) and linear regression as part of their resident assessment (30). Most of these papers assessed pre-existing smart devices and robots, which may not necessarily have been designed for a nursing home setting, leaving little by way of new developments specifically targeting nursing homes. This circumstance reveals that many IP systems are focused on applying preexisting software and hardware to the nursing home setting.

**Figure 12:**
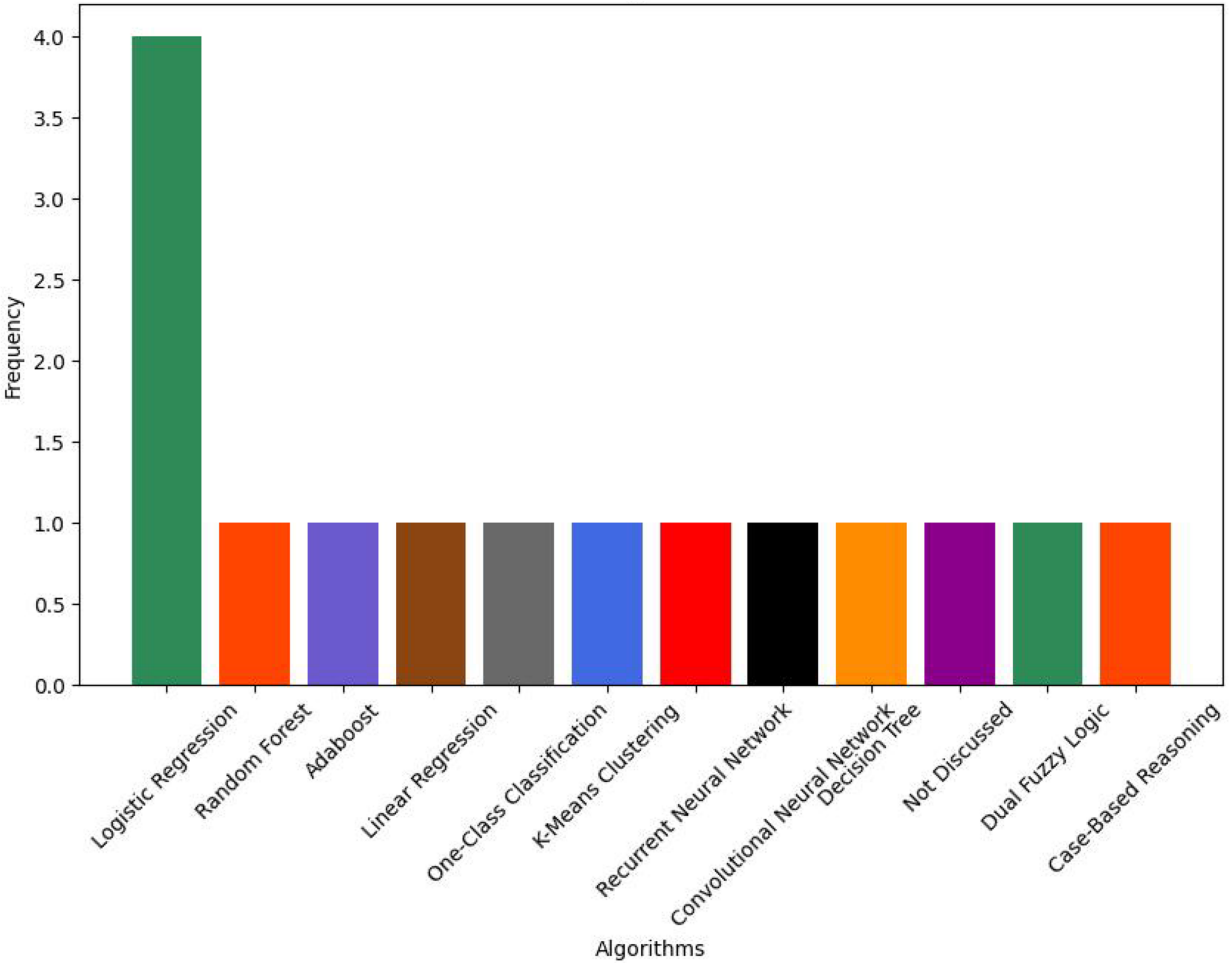
DS system ML algorithms.

The majority of DS systems in this review included some form of ML. Only one relevant study did not discuss any specific details regarding types of AI used; instead it reported user-centered exploratory qualitative research (38). All of the studies that discussed details of their AI-based implementation used supervised ML algorithms. One study also incorporated unsupervised learning into their system design (24). As shown in Figure 12, there are a vast number of different ML algorithms discussed in the literature.

These algorithms generally seem to be chosen for simplicity, conventionality, or due to the types of data available (31). For instance, all studies which used clinical data also used logistic regression (25, 31, 35, 39). Due to the wide variety of sensors used across studies, a wide variety of different algorithms are used for contrasting sensors. For example, ECG and EDA sensor data were used with Random Forest and AdaBoost algorithms, whereas BCG sensor data was used alongside one-class classification, K-means clustering, and linear regression (22, 24). Overall, there seems to be an over-reliance on supervised algorithms; much of this may be due to the limited types of data available.

As shown in Table 7, many studies, especially within Europe, seem to implement solutions with extremely small datasets (22, 24, 31, 33, 34, 38). The few studies that used bigger clinical datasets exhibited better results with similar algorithms (25, 35, 39). Whether novel insights may be uncovered from unsupervised learning using small datasets has yet to be explored. Additionally, sensor-based methods seem to be heterogeneous and may benefit from standardization to optimize algorithm choice for context-based discovery, rather than relying on traditional approaches and familiar algorithmic options.

**Table 7:** Number of study participants in each DS system study.

Study authors	Country	Participants
Delmastro et al. (22)	Italy	9
Tateno et al. (33)	Japan	16
Becker et al. (34)	Germany	16
Hayashi et al. (24)	Czech Republic	16
Bourbonnais et al. (38)	Canada	20
Miranda-Duro et al. (31)	Spain	31
Gannod et al. (39)	USA	255
Ambagtsheer et al. (35)	Australia	592
Sarwar et al. (25)	Australia	2,588

#### 3.3.3 End-user involvement

**Figure 13:**
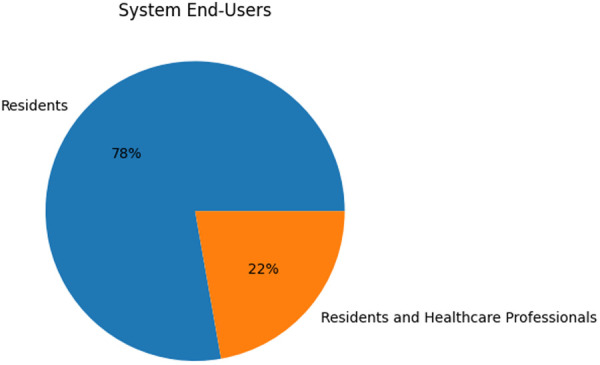
IP system end-users.

The majority of IP system research focused on observational responses within nursing homes. As shown in Figure 13, residents are the end-users of all of these systems; a small amount of the relevant literature also included caregivers as potential users.

There were only four IP studies which included residents in system evaluation (23, 27, 32, 36). As the majority of such systems are created to improve the lives of those residing in nursing homes, the residents themselves should be considered in design and evaluation processes. Additionally, the vast majority of these studies do not report feedback from healthcare professionals; only two studies reported on caregiver system evaluation (28, 29). As healthcare professionals are major stakeholders in the adoption of such technologies, there is a clear need for an increase in feedback from the same.

**Figure 14:**
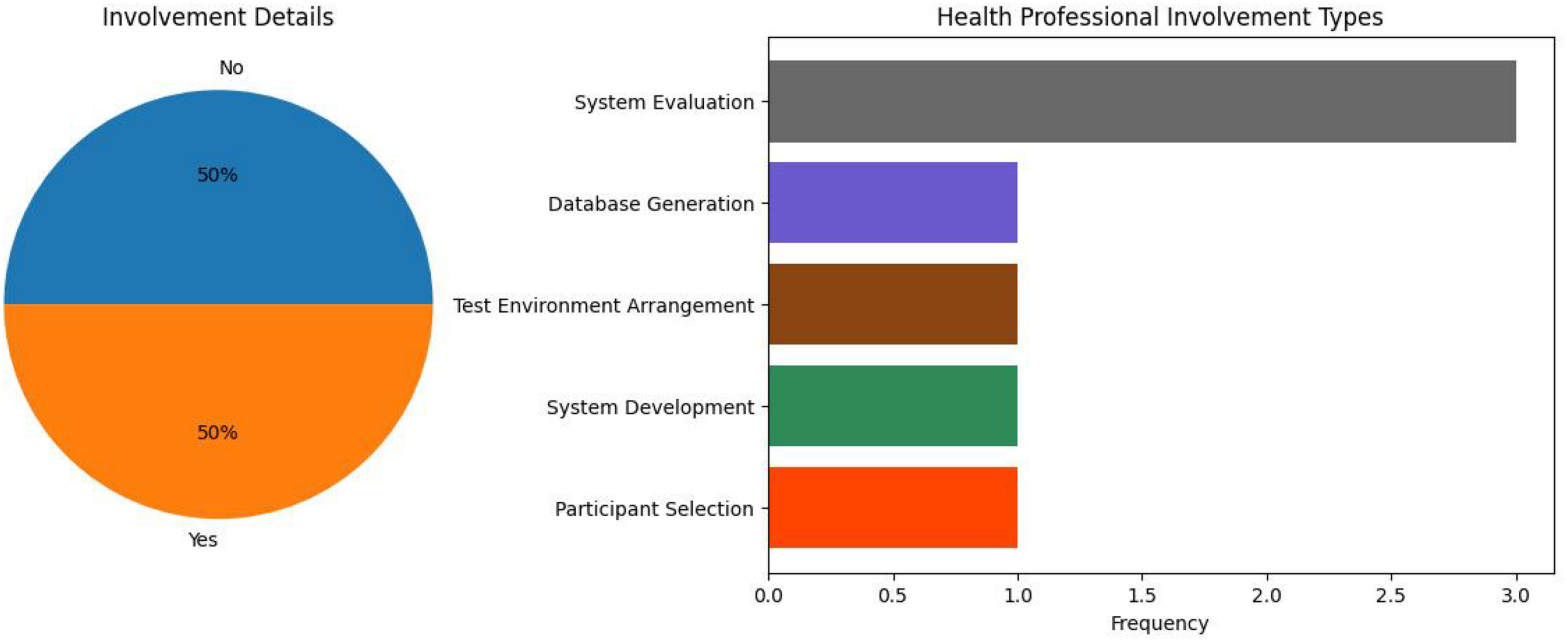
DS system health professional involvement.

100% of the end-users in DS system research were healthcare professionals. However, the majority of studies did not include healthcare professionals in their research at all. As shown in Figure 14, only two of the systems included healthcare professionals in system evaluation processes (25, 38). In all other involvements, healthcare professionals were part of simple data collection and experimental setup procedures. Furthermore, as systems primarily revolve around data collected from residents in nursing homes, resident feedback should also be of importance to researchers in the field. However, no studies incorporated resident feedback into their work.

## 4 Discussion

There seems to be a general consensus that sensors and clinical data can generate useful palliative results. However there are no standardized or common types of clinical or sensor data collected across these studies. As a result, direct comparisons between studies are difficult to create; this situation is exacerbated by the lack of clear palliative definitions across studies. Additionally, without adequate feedback from system end-users, it is not possible to assess the feasibility and acceptability of proposed interventions in future nursing homes. Overall, there are some note-worthy gaps in the literature which provide ample scope for future research.

### 4.1 PC definitions

None of the studies within this review define exactly what PC means in their research. This circumstance reflects the same ambiguity found in the broader palliative research field (7). Additionally, to the best of our knowledge, there is no non-disease-specific core outcome measure for palliative and end of life care in nursing homes. While concepts of well-being and quality of life may be loosely discussed in many publications, the lack of a distinct understanding of PC brings uncertainty to research. Studies are not directly comparable as they do not discuss the same palliative values. This inconsistency also allows such research to spread to indefinite and extreme solutions, from social applications to obscure care personalization methods. Additionally, many of the hazy and underdeveloped interpretations of PC do not seem to be informed by clinical practice, standards, or protocols. As common PC approaches are not considered in these solutions, caregivers are less likely to become familiar with new forms of palliative technology. Lack of familiarity makes such systems less likely to be accepted in future practice; caregivers want to understand the technology that is created to help them (44).

While definitions of PC are not provided and palliative outcomes for both IP and DS systems are fractured, PC is most commonly linked with improved quality of life in the literature. Most outcomes are aimed towards improving emotional and social elements of the lives of those in nursing homes. There is also an element of finality about the approaches to care discussed. These interventions are mostly about comfort and control in the last stages of life. However, providing a more concrete sense of PC across studies is difficult given the lack of discussion in the papers themselves.

Future researchers ought to include clear definitions of PC in publications in order to begin standardizing on the same. Palliative experts should be consulted to clarify what nursing home PC should be described as in technological research. Concept analysis of PC reveals the field to be quite broad; this allows many interpretations and misconceptions to co-exist in education and application (45). Core outcome measures for palliative and end of life care in nursing homes also need to be created; these measures need to factor in technological research. In order to further ideas from past projects, concrete conceptual foundations need to be built, preventing ill-conceived technologies and strengthening further work.

### 4.2 Data availability

Of those studies which used ML, there seems to be a paucity of internationally standardized data available. Only one open-source dataset could be obtained from the research publications included in this review. This constrained information landscape seems to be partially fueled by data privacy policies, such as General Data Protection Regulation (GDPR) in Europe (46). Additionally, issues surrounding health data interoperability further complicate matters in European countries (47). European DS system studies in this review feature small, unstandardized datasets with limited results; not only are findings potentially restricted to their countries of creation, they may also not be applicable outside of the nursing home in which the relevant data was collected. These types of disparate data, especially sensor data, may not be feasible for many nursing homes to collect, making such solutions impractical in common practice (48). ML usage in research with clinical data all seems to revolve around the use of conventional and familiar algorithms. This signifies that AI usage itself is stagnating within nursing home studies; the focus seems to be on applying the same methods to new data, not optimizing algorithms for standard data. Without homogeneity between clinical datasets, researchers limit themselves to the same algorithms as their counterparts; this situation hinders the use of novel approaches to valuable health information.

Comparison between European and Australian-American ML studies reveals that more data fuels more successful research. DS research from Australia and America exhibits the use of standardized datasets from multiple nursing homes; these large datasets prove themselves useful in generating more accurate algorithmic results. However, even with standardization, these findings may not be applicable outside of the countries in which they are created. Nursing documentation, which is collected across clinical settings and countries, is not adequately exploited in research (49, 50). While not standardized in themselves, nurse notes from care home documentation, could be utilized to create internationally-applicable ML models.

In general, healthcare data is not easy to access (51). Without moves to make data more accessible, future ML-based nursing home solutions may not be relevant in countries where data is not available. Anonymized datasets made openly accessible to researchers from multinational nursing homes could accelerate future research. However, in efforts to make ML-based palliative solutions, researchers must not disrespect resident values surrounding privacy and data usage. Resident data for research cannot be collected without consent due to ethical concerns. These data availability and standardization issues may take decades to fully resolve; therefore, new approaches to data generation may provide temporary solutions for present researchers.

In a manner similar to the pre-clinical investigation of drugs, future AI solutions may undergo trial processes with standard sets of generated data (52). AI itself can be used to make these synthetic datasets, such as artificial clinical note creation (53). These datasets ought to be validated by experts to guarantee pseudo-authenticity. Noise and hallucinations in the data should also be controlled. If future ML solutions were trained using this freely available information, it would make comparison between algorithmic approaches a lot more viable. Furthermore, should these datasets be created to contain information which is known to be collected internationally, such as nurse notes, future research may be pertinent to a wider range of nursing homes. These “pre-clinical” investigations may also encourage nursing homes and their residents to more openly share their data in “human” trials; subsequently, widespread adoption of standardized ML solutions may be possible.

### 4.3 Stakeholder involvement

Across studies, there is a lack of research involving both patient and health professional perspectives. Additionally, many DS systems do not include caregivers in any evaluation processes. The use of ML in monitors, analysis tools, care planners and recommender systems along with smart devices for entertainment and socialisation needs a diverse range of perspectives from both caregivers and nursing home residents. As end-users are of primary importance to system success, the lack of inclusion of caregivers and residents in design and development procedures calls the feasibility of proposed solutions into question (54).

In past research on older adult care technology and caregiver perspectives, it was found that nurses want to be involved in the development of solutions; there is an emphasis placed on including stakeholders such that future systems are designed based on need, not on the availability of certain technologies (44). Furthermore, residents’ opinions should also be included in system analysis due to their inherent involvement in the use of such technologies (55). None of the studies included in this review report on the inclusion of residents’ family members in research processes; family members often act as proxies for residents in nursing homes and can be crucial in PC discussions (56).

It is important to involve these stakeholders in the design of such technology to understand what tools are most needed in PC settings. Additionally, it is important to consider what impact these systems have on those interacting with them. If a PC system is not perceived positively by those it aims to help, then it is in need of improvement. It cannot be improved effectively without help from those who are face-to-face with PC scenarios on a daily basis. While ethical issues can make the inclusion of all parties difficult, future work should aim to consider as many stakeholders as possible in the design and implementation of AI-based systems.

### 4.4 Alternative technologies

Caregivers want to have more time to spend with their residents (44). Documentation is time-consuming; logging information in nursing homes can outweigh the time caregivers have to spend with residents (44). Text-centered solutions have been cited as promising areas of exploration within PC research (57). AI has the potential to alleviate the nurse’s workload while increasing the amount of person-centered care a resident receives (58). Additionally, caregivers do not want AI substitutes, they want collaborators; all aspects of care should not be replaced with machines (58). While many studies in this review focus on the use of AI for human-centered processes, such as socialization and qualitative interaction, there is a lack of research into the use of AI for task automation, such as documentation creation.

Similar to findings reported by Cunha et al., we believe that an integrated approach to care, involving multiple technologies, is possible in the future (59). There is a clear divide between research into IP systems and DS systems in the literature. Niche systems with narrow scope ought to be integrated into a wider care landscape in order to be practical. IP components beyond the scope of robots and speakers, such as glasses, can be explored in conjunction with decision support (DS) intelligent physical (IP) methods (55). These devices could be utilised as new tools to aid nurses in everyday activities, such as documentation and clinical data generation.

Privacy is a clear issue in nursing homes; surveillance-based technologies have been discouraged in multiple studies due to the potential to violate resident wishes (38, 58). While many studies in this review focus on the use of AI in sensitive everyday care scenarios, no study considers the application of smart devices or ML in less-private nursing home settings, such as family meetings. Family meetings are a crucial aspect of PC in nursing homes; they help define resident wishes and needs, ensuring all relevant parties are in communication with each other. These meetings enable improved quality of life for residents (60). Subsequent documentation of the same is also extremely important when updating advanced care plans for patients (61). Advanced care planning (ACP) is the process of recording patient preferences concerning goals of care (62). However, accurate and appropriate documentation of the same takes a lot of caregivers’ time (60, 63). Smart technologies such as speakers, glasses, and other audio-visual technologies could be utilised to transcribe, annotate, and document such meetings in an efficient and privacy-centred manner.

### 4.5 Limitations

No literature review can exist without its own set of limitations. For this review, we focused on six databases pertinent to the field of computer science. In limiting ourselves to these sources, we potentially exclude publications from alternative locations, such as those from health-focused databases. Additionally, although all authors have been involved in a comprehensive assessment of this review, the primary author conducted the majority of the study selection procedure; this circumstance was due to resource constraints. While the use of a pre-existing protocol limits some potential for selection bias, there may still be bias present.

We chose to use stringent inclusion and exclusion criteria. While these criteria ensure only articles of the highest quality, relevance, and validity are included in our analysis, there may be useful information in excluded articles. Articles not in English and articles prior to 2017 may provide a different overview of the field. By relaxing the impact criterion, a broader view of the research landscape may be obtained. Furthermore, only conference and journal articles are considered, limiting the breadth of review results; prospective reviews could consider book chapters and other forms of academic literature in their analysis.

## 5 Conclusions

This PRISMA-ScR scoping review was undertaken to investigate the current state of research on AI usage for PC in nursing homes (16). Our findings are based on 19 research articles; our discussion centered on palliative definitions, data accessibility measures, stakeholder involvement, and future technological directions.

Bibliometric analysis revealed that European studies were most prevalent; North American, Australian and Asian studies also feature. There is a notable paucity of research from South American and African studies, indicating that people from these locations have unmet PC needs or that there is not comparable research activity in these regions. No authors were found to be particularly prevalent within this field. While indicating that a diverse range of views are incorporated into our review, this finding also reveals the fragmented research landscape.

Key nursing home stakeholders are not consistently involved in system design and evaluation processes. Healthcare professionals, residents, and relevant family members should be involved in research to ensure any outputs from studies are feasible in practice. Additionally, palliative outcomes from relevant materials are varied and definitions of PC are not mentioned; this situation reflects the confusion found in the general palliative research area (8). In order to compare future studies without ambiguity, definitions of PC need to be included in publications. PC experts should be consulted, or at least referenced, in order to avoid disparate solutions.

Our findings indicate that novel ML approaches to clinical data for DS systems need to be investigated; researchers ought to move beyond the use of simple regression models. Palliative research involving ML may be stagnating due to the lack of data available. Data sharing procedures could be established to accelerate and instigate further research in the field. Data issues may take years to resolve; artificial data may be used to convey the advantages of AI usage in nursing homes, thus encouraging future facilities and their residents to share their data for further research. There is also scope to investigate internationally-collected types of data, such as nurse notes and free-text documentation. Additionally, sensor-based DS research seems to be heterogeneous, unstandardized, and invasive; this finding indicates that such technologies are inapplicable in many facilities and unsuitable for present nursing home environments.

Our study also finds that there is a clear divide between research focusing on IP and DS systems. Integrated DS and IP systems need to be developed. IP systems are mainly focused on socially assistive robots; other devices, such as smart glasses, have not been adequately explored. Task automation tools involving elements of both IP and DS systems have yet to be investigated.

The main contribution of this study is a review of AI systems for PC in nursing homes. Our findings indicate that there is ample room for research into data availability solutions, integrated IP and DS systems, and stakeholder involvement. Combining multiple forms of data collection and technology together may be possible in future systems. Clearer definitions of PC in subsequent research are also needed in order to be able to accurately compare studies. Additionally, future research should include healthcare professionals, residents’ families, and the residents themselves in the system design and assessment processes. Alternative technologies focused on everyday tasks may be developed in consultation with these stakeholders in order to increase the amount of person-centered care provided by caregivers. By addressing these challenges, it will be possible to create a stronger palliative landscape for our aging population using AI.

## Data Availability

The datasets presented in this study can be found in online repositories. The names of the repository/repositories and accession number(s) can be found below: https://github.com/isabel-ronan/reviewNursingHomeAI.
