# Supplementary material for: Artificially intelligent nursing homes: a scoping review of palliative care interventions
Source: Front Digit Health. 2025 Feb 11;7:1484304. doi: 10.3389/fdgth.2025.1484304 (PMC11851530; doi:10.3389/fdgth.2025.1484304)
Supplement: Supplementary file 1 [file Datasheet1.pdf]

Article Citation	Main Exclusion Reason
Yousaf K, Mehmood Z, Awan IA, Saba T, Alharbey R, Qadah T, et al. A comprehensive study of mobile-health based assistive technology for the healthcare of dementia and Alzheimer's disease (AD). Health Care Manag Sci. 2020 Jun;23(2):287–309.	Not Relevant to My Research
Vinay R, Biller-Andorno N. A critical analysis of national dementia care guidances. Health Policy. 2023 Apr;130:104736.	Not Relevant to My Research
Harding AJE, Doherty J, Bavelaar L, Walshe C, Preston N, Kaasalainen S, et al. A family carer decision support intervention for people with advanced dementia residing in a nursing home: a study protocol for an international advance care planning intervention (mySupport study). BMC Geriatrics. 2022 Oct 26;22(1):822.	Not Relevant to My Research
Vikström S, Grönstedt HK, Cederholm T, Franzén E, Seiger Å, Faxén-Irving G, et al. A health concept with a social potential: an interview study with nursing home residents. BMC Geriatrics. 2020 Sep 4;20(1):324.	Not Relevant to My Research, Limitations Not Clearly Discussed
Lee HTS, Chen TR, Yang CL, Chiu TY, Hu WY. Action research study on advance care planning for residents and their families in the long-term care facility. BMC Palliative Care. 2019 Nov 5;18(1):95.	Not Relevant to My Research
Raychoudhury M, Yu H, Kiper J. ActiviSee: Activity Transition Detection for Human Users through Wearable Sensor-augmented Glasses. In 2022. p. 371–8.	Not Relevant to My Research, Limitations Not Clearly Discussed
Cohen-Mansfield J, Muff A, Meschiany G, Lev-Ari S. Adequacy of Web-Based Activities as a Substitute for In-Person Activities for Older Persons During the COVID-19 Pandemic: Survey Study. Journal of Medical Internet Research. 2021 Jan 22;23(1):e25848.	Not Relevant to My Research
Han X, Zhou X, Tan B, Jiao L, Zhang R. Ai-based next-generation sensors for enhanced rehabilitation monitoring and analysis. Measurement. 2023 Dec 1;223:113758.	Not Relevant to My Research
Yamada K. An acquaintance with an aging society. Social Sciences. 2019;8(4).	Not Relevant to My Research, Limitations Not Clearly Discussed, Methodology Not Clearly Outlined

Jarvis MA, Padmanabhanunni A, Chipps J. An evaluation of a low-intensity cognitive behavioral therapy mhealth-supported intervention to reduce loneliness in older people. International Journal of Environmental Research and Public Health. 2019;16(7).	Not Relevant to My Research
Guimarães V, Oliveira E, Carvalho A, Cardoso N, Emerich J, Dumoulin C, et al. An exergame solution for personalized multicomponent training in older adults. Applied Sciences (Switzerland). 2021;11(17).	Not Relevant to My Research, Limitations Not Clearly Discussed
Leung MY, Sieh L, Yin R. An integrated model for luminous environment and quality of life of older people in care and attention homes. Building and Environment. 2023;244.	Not Relevant to My Research
Frennert S. Approaches to welfare technology in municipal eldercare. Journal of Technology in Human Services. 2020;38(3):226–46.	Not Relevant to My Research
Skuban-Eiseler T, Orzechowski M, Denkinger M, Kocar TD, Leinert C, Steger F. Artificial Intelligence–Based Clinical Decision Support Systems in Geriatrics: An Ethical Analysis. Journal of the American Medical Directors Association. 2023;24(9):1271-1276.e4.	Not Relevant to My Research, Results Not Clearly Described
Coco K, Kangasniemi M, Rantanen T. Care Personnel's Attitudes and Fears Toward Care Robots in Elderly Care: A Comparison of Data from the Care Personnel in Finland and Japan. Journal of Nursing Scholarship. 2018;50(6):634–44.	Not Relevant to My Research
Vallès-Peris N, Domènech M. Care robots for the common good: ethics as politics. Humanit Soc Sci Commun. 2023 Jun 21;10(1):1–6.	Methodology Not Clearly Outlined, Limitations Not Clearly Discussed, Not Relevant to My Research
Rejula V, Anitha J, Belfin RV, Peter JD. Chronic Pain Treatment and Digital Health Era-An Opinion. Frontiers in Public Health. 2021;9.	Not Relevant to My Research, Limitations Not Clearly Discussed
Bernabeu-Wittel M, Ternero-Vega JE, Díaz-Jiménez P, Conde-Guzmán C, Nieto-Martín MD, Moreno-Gaviño L, et al. Death risk stratification in elderly patients with covid-19. A comparative cohort study in nursing homes outbreaks. Archives of Gerontology and Geriatrics. 2020;91.	Not Relevant to My Research
Suzuki M, Yamamoto R, Ishiguro Y, Sasaki H, Kotaki H. Deep learning prediction of falls among nursing home residents with Alzheimer's disease. Geriatrics & Gerontology International. 2020;20(6):589–94.	Not Relevant to My Research

Almalki M, Alsulami MH, Alshdadi AA, Almuayqil SN, Alsaqer MS, Atkins AS, et al. Delivering Digital Healthcare for Elderly: A Holistic Framework for the Adoption of Ambient Assisted Living. International Journal of Environmental Research and Public Health. 2022;19(24).	Not Relevant to My Research
Huang Y, Xu T, Yang Q, Pan C, Zhan L, Chen H, et al. Demand prediction of medical services in home and community-based services for older adults in China using machine learning. Frontiers in Public Health. 2023;11.	Not Relevant to My Research
Stegner L, Mutlu B. Designing for Caregiving: Integrating Robotic Assistance in Senior Living Communities. In 2022. p. 1934–47.	Not Relevant to My Research
Dijkman BL, Hirjaba M, Wang W, Palovaara M, Annen M, Varik M, et al. Developing a competence framework for gerontological nursing in China: a two-phase research design including a needs analysis and verification study. BMC Nursing. 2022 Oct 26;21(1):285.	Not Relevant to My Research
Ide H, Suwa S, Akuta Y, Kodate N, Tsujimura M, Ishimaru M, et al. Developing a model to explain users' ethical perceptions regarding the use of care robots in home care: A cross-sectional study in Ireland, Finland, and Japan. Archives of Gerontology and Geriatrics. 2024;116.	Not Relevant to My Research
Fares N, Sherratt RS, Elhajj IH. Directing and orienting ict healthcare solutions to address the needs of the aging population. Healthcare (Switzerland). 2021;9(2).	Not Relevant to My Research
Mu J, Kang J, Sui Z. Effect of music in large activity spaces on the perceptions and behaviours of older adults in China. Applied Acoustics. 2022;188.	Not Relevant to My Research
Zhang Y, Gupta A, Nicholson S, Li J. Elevated end-of-life spending: A new measure of potentially wasteful health care spending at the end of life. Health Services Research. 2023;58(1):186–94.	Not Relevant to My Research
Dupont C, Monnet F, Pivodic L, Vleminck AD, Audenhove CV, Van den Block L, et al. Evaluating an advance care planning website for people with dementia and their caregivers: Protocol for a mixed method study. Digital Health. 2023;9.	Not Relevant to My Research
Fitter NT, Mohan M, Kuchenbecker KJ, Johnson MJ. Exercising with Baxter: Preliminary support for assistive social-physical human-robot interaction. Journal of NeuroEngineering and Rehabilitation. 2020;17(1).	Not Relevant to My Research

Miller VJ, Roark EM, Fields NL, Cronley C. Experiences with Technology Amongst an International Sample of Older Adults: Results from a Qualitative Interpretive Meta-Synthesis. British Journal of Social Work. 2021;51(4):1332–53.	Not Relevant to My Research
Gutiérrez F, Htun NN, Vanden Abeele V, De Croon R, Verbert K. Explaining Call Recommendations in Nursing Homes: A User-Centered Design Approach for Interacting with Knowledge-Based Health Decision Support Systems. In: 27th International Conference on Intelligent User Interfaces [Internet]. New York, NY, USA: Association for Computing Machinery; 2022. p. 162–72. (IUI ’22). Available from: https://doi.org/10.1145/3490099.3511158	Not Relevant to My Research
Alexander GL, Deroche C, Powell K, Mosa ASM, Popejoy L, Koopman R. Forecasting Content and Stage in a Nursing Home Information Technology Maturity Instrument Using a Delphi Method. J Med Syst. 2020 Feb 5;44(3):60.	Not Relevant to My Research
Suikkala A, Tohmola A, Rahko EK, Hökkä M. Future palliative competence needs – a qualitative study of physicians’ and registered nurses’ views. BMC Medical Education. 2021 Nov 17;21(1):585.	Not Relevant to My Research
Maiti D, Awasthi A. ICT Exposure and the Level of Wellbeing and Progress: A Cross Country Analysis. Social Indicators Research. 2020;147(1):311–43.	Not Relevant to My Research
Attwood D, Vafidis J, Boorer J, Long S, Ellis W, Earley M, et al. IT-assisted comprehensive geriatric assessment for residents in care homes: quasi-experimental longitudinal study. BMC Geriatrics. 2024 Mar 19;24(1):269.	Not Relevant to My Research
Heinzen EP, Wilson PM, Storlie CB, Demuth GO, Asai SW, Schaeferle GM, et al. Impact of a machine learning algorithm on time to palliative care in a primary care population: protocol for a stepped-wedge pragmatic randomized trial. BMC Palliative Care. 2023 Feb 3;22(1):9.	Not Relevant to My Research
Akram A, Nicosia F, Lee J, Lee M, Martin L, Martinez S, et al. Implementation of an integrative movement program for residents with dementia in a VA nursing home. BMC Geriatrics. 2021 Oct 27;21(1):607.	Not Relevant to My Research
Thoolen M, Toso F, Peek STM, Lu Y, Brankaert R. LivingMoments: Bespoke Social Communication for People living with Dementia and their Relatives. In 2022.	Not Relevant to My Research

Viana JN, Pilbeam C, Howard M, Scholz B, Ge Z, Fisser C, et al. Maintaining High-Touch in High-Tech Digital Health Monitoring and Multi-Omics Prognostication: Ethical, Equity, and Societal Considerations in Precision Health for Palliative Care. OMICS A Journal of Integrative Biology. 2023;27(10):461–73.	Not Relevant to My Research
Sen K, Prybutok V, Prybutok G, Senn W. Mechanisms of Social Interaction and Virtual Connections as Strong Predictors of Wellbeing of Older Adults. Healthcare (Switzerland). 2022;10(3).	Not Relevant to My Research
Mathkor DM, Mathkor N, Bassfar Z, Bantun F, Slama P, Ahmad F, et al. Multirole of the internet of medical things (IoMT) in biomedical systems for managing smart healthcare systems: An overview of current and future innovative trends. Journal of Infection and Public Health. 2024;17(4):559–72.	Limitations Not Clearly Discussed
Zhang L, Han Y, Fang Y. Non-human and human service efficiency of long-term care facilities in China. Frontiers in Public Health. 2023;11.	Not Relevant to My Research
Zhao W, Kelly RM, Rogerson MJ, Waycott J. Older Adults Using Technology for Meaningful Activities During COVID-19: An Analysis Through the Lens of Self-Determination Theory. In 2023.	Not Relevant to My Research
Ott T, Heckel M, Öhl N, Steigleder T, Albrecht NC, Ostgathe C, et al. Palliative care and new technologies. The use of smart sensor technologies and its impact on the Total Care principle. BMC Palliative Care. 2023 Apr 26;22(1):50.	Not Relevant to My Research
Sánchez-Cárdenas MA, Pourghazian N, Garralda E, van Steijn D, Slama S, Benítez E, et al. Palliative care in the Eastern Mediterranean: comparative analysis using specific indicators. BMC Palliative Care. 2022 Oct 3;21(1):168.	Not Relevant to My Research
Harrison SL, Dyer SM, Laver KE, Milte RK, Fleming R, Crotty M. Physical environmental designs in residential care to improve quality of life of older people. Cochrane Database of Systematic Reviews. 2022;2022(3).	Not Relevant to My Research
Blanes-Selva V, Doñate-Martínez A, Linklater G, Garcés-Ferrer J, García-Gómez JM. Responsive and minimalist app based on explainable ai to assess palliative care needs during bedside consultations on older patients. Sustainability (Switzerland). 2021;13(17).	Not Relevant to My Research

Li Y, Bai L, Mao Y, Ren H, Qiao Y, Tong X, et al. Rethinking pain communication of patients with Alzheimer's disease through E-textile interaction design. Frontiers in Physiology. 2023;14.	Not Relevant to My Research
Chandra A, Takahashi PY, McCoy RG, Thorsteinsdottir B, Hanson GJ, Chaudhry R, et al. Risk Prediction Model for 6-Month Mortality for Patients Discharged to Skilled Nursing Facilities. Journal of the American Medical Directors Association. 2022 Aug 1;23(8):1403–8.	Not Relevant to My Research
Nwosu AC, Sturgeon B, McGlinchey T, Goodwin CDG, Behera A, Mason S, et al. Robotic technology for palliative and supportive care: Strengths, weaknesses, opportunities and threats. Palliative Medicine. 2019;33(8):1106–13.	Not Relevant to My Research
Pu L, Bakker C, Appelhof B, Zwijsen SA, Teerenstra S, Smalbrugge M, et al. The Course of Quality of Life and Its Predictors in Nursing Home Residents With Young-Onset Dementia. Journal of the American Medical Directors Association. 2021;22(7):1456-1464.e1.	Not Relevant to My Research
Koh WQ, Vandemeulebroucke T, Gastmans C, Miranda R, Van den Block L. The ethics of pet robots in dementia care settings: Care professionals' and organisational leaders' ethical intuitions. Frontiers in Psychiatry. 2023;14.	Not Relevant to My Research
Zhao Y, Sazlina SG, Rokhani FZ, Chinna K, Su J, Chew BH. The expectations and acceptability of a smart nursing home model among Chinese older adults: a mixed methods study. BMC Nursing. 2024 Jan 13;23(1):40.	Not Relevant to My Research
Toscani F, Finetti S, Giunco F, Basso I, Rosa D, Pettenati F, et al. The last week of life of nursing home residents with advanced dementia: a retrospective study. BMC Palliative Care. 2019 Dec 27;18(1):117.	Not Relevant to My Research
Łukasik S, Tobis S, Suwalska J, Łojko D, Napierała M, Proch M, et al. The role of socially assistive robots in the care of older people: To assist in cognitive training, to remind or to accompany? Sustainability (Switzerland). 2021;13(18).	Not Relevant to My Research
Su D, Zhang X, He K, Chen Y. Use of machine learning approach to predict depression in the elderly in China: A longitudinal study. Journal of Affective Disorders. 2021;282:289–98.	Not Relevant to My Research

Gühne U, Dorow M, Grothe J, Stein J, Löbner M, Dams J, et al. Valuing end-of-life care: translation and content validation of the ICECAP-SCM measure. BMC Palliative Care. 2021 Feb 8;20(1):29.	Not Relevant to My Research
Davila H, Ng W, Akosionu O, Thao MS, Skarphol T, Virnig BA, et al. Why Men Fare Worse: A Mixed-Methods Study Examining Gender Differences in Nursing Home Resident Quality of Life. Gerontologist. 2022;62(9):1347–58.	Not Relevant to My Research
Wróbel-Lachowska M, Dominiak J, Woźniak MP, Bartłomiejczyk N, Diethei D, Wysokińska A, et al. 'That's when I put it on': stakeholder perspectives in large-scale remote health monitoring for older adults. Personal and Ubiquitous Computing. 2023;27(6):2193–210.	Not Relevant to My Research
Walter J, Tufman A, Holle R, Schwarzkopf L. "Age matters"—German claims data indicate disparities in lung cancer care between elderly and young patients. PLoS ONE. 2019;14(6).	Not Relevant to My Research
Lound A, Bruton J, Jones K, Shah N, Williams B, Gross J, et al. "I'd rather wait and see what's around the corner": A multi-perspective qualitative study of treatment escalation planning in frailty. PLoS ONE. 2023;18(9 September).	Methodology Not Clearly Outlined
